# Immunophenotypic evolution of blast populations in pediatric acute myeloid leukemia

**DOI:** 10.1590/S1679-45082016AI3516

**Published:** 2016

**Authors:** Welbert Oliveira Pereira, Rodolfo Patussi Correia, Nelson Hamerschlak, Nydia Strachman Bacal, Paulo Vidal Campregher

**Affiliations:** 1 Hospital Israelita Albert Einstein, São Paulo, SP, Brazil.

Acute myeloid leukemia (AML) results from accumulation of abnormal blasts in the bone marrow, interfering with hematopoiesis.^(^ ^1^ ^)^ Multiparametric flow cytometry is an important and well-known tool for AML diagnosis and for monitoring of minimal residual disease.^(^ ^2^ ^)^ However, this technique is still poorly explored to elucidate clonal evolution of the leukemia. We present laboratory observations of a pediatric AML case whose multiparametric flow cytometry data provided evidences of clonal history of leukemia from the diagnosis through relapses episodes.

A 7-year-old patient, diagnosed as AML with maturation, with complex karyotype, wild type FLT3, negative for 11q23, inv(16), t(9;22), and for t(8;21). Flow cytometry analysis showed two distinct subpopulations of CD34(+) blasts in the bone marrow, with 72.5% of blasts expressing CD34/CD33/CD117/CD11b/CD56, and 16.1% expressing the same markers with additional CD7. After treatment, minimal residual disease was monitored until the achievement of immunophenotypic remission. Six months after starting therapy, the patient relapsed with 75.1% of blasts in the bone marrow, divided into three separate subpopulations: 42% expressing CD34/CD117/CD11b/CD56/CD7 (this population persisted from the diagnosis), 22.5% expressing high levels of the B-cell marker CD19, and a third one expressing lower levels of CD19 (10.5% of the cells). Unlike the diagnosis profile, all clones were CD7(+) (Figure 1A to 1C). The treatment reduced the leukemia burden to 0.04% and unrelated-donor bone marrow transplantation (matched unrelated donor) was performed. Three months after bone marrow transplantation, the patient relapsed with 62.9% of blasts in the bone marrow, in which all expressed CD19 but represented in two distinct immunophenotypic populations: CD34/CD33/CD117/CD11b/CD19/CD56/CD7 (40%) and another lacking CD56 and CD7 (22.9%) (Figure 1D). After additional chemotherapy, the patient had persistent disease. In the last evaluation we found 39.7% of blasts in the bone marrow expressing CD7/CD56/CD19 and cytoplasmic CD3. A small fraction (5%) of these cells presented lower levels of CD19, suggestive of a distinct subpopulation (Figure 1E). Interestingly, both populations lacked myeloperoxidase expression, although have maintained the other original myeloid markers (CD33/CD117/CD11b). Monitoring the immunophenotype of the blasts during the progression of the AML allowed us to assume the clone history of the disease, which revealed the complexity of this case (Figure 2). Under chemotherapy, we observed the complete disappearance of some blast populations, whereas others persisted and originated new subpopulations in the following relapse.

**Figure 1:**
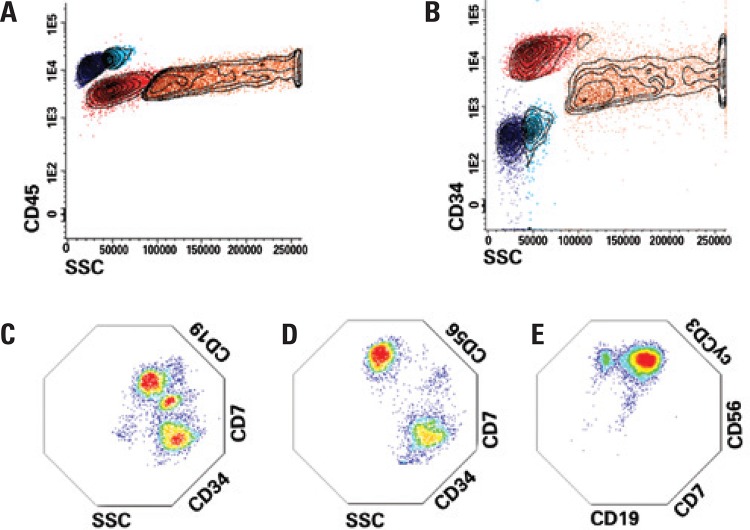
Multiparametric flow cytometry immunophenotyping reveals the diversity of blast subpopulation in acute myeloid leukemia. (A and B) Blast cells were gated according to the low expression of CD45 and high expression of CD34 (red population). Lymphocytes (blue population), monocytes (aqua population) and granulocytes (orange population) were also gated. (C) Multi parametric flow cytometry showed the presence of three different subpopulations of CD7-positive blasts in the first relapse: one CD19-negative and two distinct CD19-positive. (D) Detection of two subpopulations of blasts at the second relapse: CD7+/CD56+ and CD7-/CD56-. (E) Multi parametric flow cytometry showed the presence of only CD19+/CD7+/CD56+/CD3+ blasts. The analysis was performed using the Infinicyt software (Cytognos, Salamanca, Spain)

**Figure 2:**
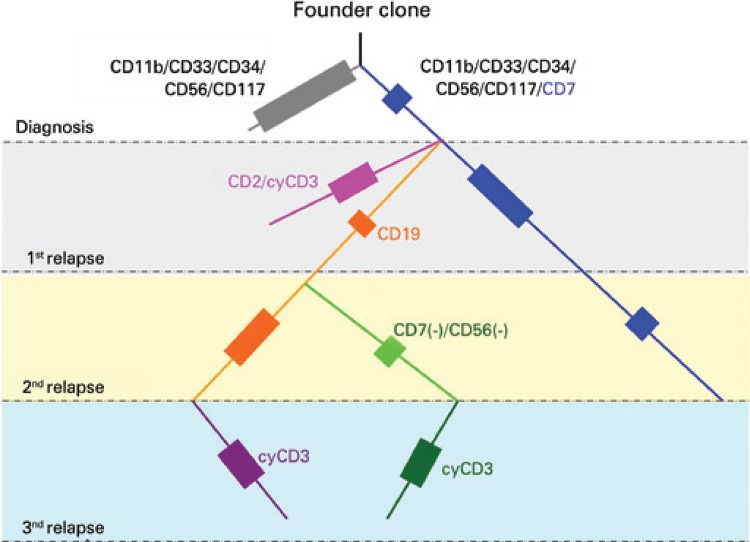
Scheme of clonal evolution and relatedness among populations in the acute myeloid leukemia inferred from immunophenotyping data. The patients were diagnosed with two populations of blasts (gray and blue), which we supposed derived from a common founder clone. At the first relapse, we detected three different subpopulations of blasts. Beside the persistent population, that expanded in frequency (blue), the other two populations presented CD19 and most likely originated from the blue population considering the expression of CD7. At the second relapse, the CD19-positive population (orange) persisted and derived a new subclone lacking CD7 and CD56. The treatment approaches seemed to be efficient to extinct the blue and pink populations. In the last relapse, we observe two subpopulations of blasts. According to the levels of CD19 expression, and the presence of CD7 and CD56, these clones derived from the persistent CD19low and CD19high populations previously observed. The colors of the lines and boxes illustrate the different subclones identified in the course of the disease. The size of the boxes indicates the frequency of these populations in each stage. The new (additional or lacked) markers that characterized the next generation of leukemic clones are highlighted with the same color of the population line. The dotted line indicates the hypothetical presence of the population. The end of the lines indicates the extinction of that population in the history of the disease

Although the gold standard for clonality studies should include molecular analysis, which was not available in this case, the multiparametric flow cytometry yielded valuable information regarding the plasticity of the blasts and the clonal evolution of AML.

## References

[1] Estey EH (2013). Acute myeloid leukemia: 2013 update on risk-stratification and management. Am J Hematol.

[2] Buccisano F, Maurillo L, Del Principe MI, Del Poeta G, Sconocchia G, Lo-Coco F (2012). Prognostic and therapeutic implications of minimal residual disease detection in acute myeloid leukemia. Blood.

